# In Vitro Anticancer Screening, Molecular Docking and Antimicrobial Studies of Triazole-Based Nickel(II) Metal Complexes

**DOI:** 10.3390/molecules27196548

**Published:** 2022-10-03

**Authors:** Sachin A. Deodware, Umesh B. Barache, Pratibha C. Dhale, Kundalkesha D. Gaikwad, Chandan Shivamallu, Panchsheela A. Ubale, Ali A. Shati, Mohammad Y. Alfaifi, Serag Eldin I. Elbehairi, Raghu Ram Achar, Ekaterina Silina, Victor Stupin, Juan Frau, Norma Flores-Holguín, Shashikant H. Gaikwad, Shiva Prasad Kollur, Daniel Glossman-Mitnik

**Affiliations:** 1 Arts, Science and Commerce College, Naldurg, Osmanabad 413 602, Maharashtra, India; 2 Chemistry Research Laboratory, Department of Chemistry, Shri Shivaji Mahavidyalaya, Solapur 413 411, Maharashtra, India; 3 School of Chemical Sciences, Punyashlok Ahilyadevi Holkar Solapur University, Solapur 413 255, Maharashtra, India; 4 Department of Biotechnology and Bioinformatics, School of Life Sciences, JSS Academy of Higher Education and Research, Mysuru 570 015, Karnataka, India; 5 Department of Chemistry, N. K. Orchid College of Engineering and Technology, Solapur 413 002, Maharashtra, India; 6 Biology Department, Faculty of Sciences, King Khalid University, Abha 61421, Saudi Arabia; 7 Cell Culture Lab, Egyptian Organization for Biological Products and Vaccines (VACSERA Holding Company), 51 Wezaret El-Zeraa St., Agouza, Giza 12311, Egypt; 8 Division of Biochemistry, School of Life Sciences, JSS Academy of Higher Education and Research, Mysuru 570 015, Karnataka, India; 9 Department of Hospital Surgery, N.I. Pirogov Russian National Research Medical University (RNRMU), 117997 Moscow, Russia; 10 Departament de Química, Facultat de Ciènces, Universitat de les Illes Balears, E-07122 Palma de Malllorca, Spain; 11 Laboratorio Virtual NANOCOSMOS, Departamento de Medio Ambiente y Energía, Centro de Investigación en Materiales Avanzados, Chihuahua 31136, Chih, Mexico; 12 School of Physical Sciences, Amrita Vishwa Vidyapeetham, Mysuru Campus, Mysuru 570 026, Karnataka, India

**Keywords:** Nickel(II) complexes, molecular docking, anticancer screening, antimicrobial studies, conceptual DFT

## Abstract

Herein we describe the synthesis of a series of nickel(II) complexes (C1–C3) with Schiff bases (HL1–HL3) derived from 4-amino-5-mercapto-3-methyl-1,2,4-triazole and ortho/meta/para-nitrobenzaldehyde having composition [Ni(L)_2_(H_2_O)_2_]. The obtained ligands and their complexes were characterized using physico-chemical techniques viz., elemental analysis, magnetic moment study, spectral (electronic, FT-IR, 1H-NMR) and thermal analysis. The elemental analysis and spectral analysis revealed that Schiff bases behave as monoanionic bidentate ligands towards the Ni(II) ion. Whereas, the magnetic moment study suggested the octahedral geometry of all the Ni(II) complexes. The thermal behavior of the complexes has been studied by thermogravimetric analysis and agrees well with the composition of complexes. Further, the biological activities such as antimicrobial and antifungal studies of the Schiff bases and Ni(II) complexes have been screened against bacterial species (*Staphylococcus aureus* and *Pseudomonas aeruginosa*) and fungal species (*Aspergillus niger* and *Candida albicans*) activity by MIC method, the results of which revealed that metal complexes exhibited significant antimicrobial activities than their respective ligands against the tested microbial species. Furthermore, the molecular docking technique was employed to investigate the active sites of the selected protein, which indeed helped us to screen the potential anticancer agents among the synthesized ligand and complexes. Further, these compounds have been screened for their in vitro anticancer activity using OVCAR-3 cell line. The results revealed that the complexes are more active than the ligands.

## 1. Introduction

In 1965, Rosenburg discovered a platinum complex, cisplatin, which establish a revolution in the treatment of cancer. After this discovery, there is a considerable increase in the use of metal complexes in the treatment of cancer. Recently, study associated with metal containing drugs, showed promising biological activities [1]. A literature survey revealed that metal complexes synthesized from chelating agent and transition metal salts exhibit enhanced physico-chemical and pharmacological properties [2,3,4]. Heteroatoms of chelating agent on reaction with positively charged metal ions produce complexes with well-defined geometries that can interact with biomolecules [5].

There are ample triazole-based derivatives are available as medicines [6]. Moreover, they are also used as agrochemical [7], supramolecular [8], electrochemical [9], corrosion retardant [10]. Most important and potential drug-like behavior of triazole-based compounds include antitumor [11], antimalarial [12], antifungal [13], antibacterial [14], antidiabetic [15] and anticonvulsant [16]. The 1,2,4-triazole-derived Schiff base ligands were demonstrated to coordinate with transition metal ions in a multidentate fashion, which also includes N-atom of the triazolyl group. The effect of complexation of pyridyl-containing inverse chelating groups can be described by the enhanced electrophilicity of the metal centre, which is caused by the coordination with the p-acceptor pyridyl group, which consequently enables coordination, even of the less-electron-rich N-atom of the 1,2,4-triazole ring [17].

The coordination compounds of 1,2,4-triazoles have a considerable interest because of their brilliant coordination potential and diverse pharmacological properties [18,19,20,21,22,23,24,25,26]. The presence of an electron withdrawing group like -NO_2_ on aromatic ring showed improved antimicrobial activity [27,28]. Due to their rich and all around coordination mode, we applied some triazole derivatives for the determination of trace amounts of precious metals like Cu(II), Pd(II), Au(III), toxic metals like Se(IV), Te(V), Bi(III) and Cr(VI) [29,30,31,32,33,34,35]. There are also some known drugs containing 1,2,4-triazole moiety like Letrozole, Anastrozole [36], Trazodone [37,38]. Literature survey reveals that, no work has been reported on the synthesis of Ni(II) complexes derived from 4-amino-5-mercapto-3-methyl-1,2,4-triazole and 2/3/4-nitrobenzaldehyde. Recently, in 2017 we reported [38,39] the synthesis and characterization of a series of cobalt, nickel and copper complexes of bidentate Schiff base derived from the condensation reaction of 4-amino-5-mercapto-3-methyl-1,2,4-triazole with 2-nitrobenzaldehyde and their utilization as an anticancer agent. Interestingly, it was found that Schiff base and its Co(II), Ni(II) and Cu(II) complexes exhibit excellent activity against breast cancer cell line MCF-7. Such results prompted us to design new compounds and aiming to get new anticancer agents.

Novel Schiff bases (HL_1_–HL_3_) and their nickel(II) complexes (C_1_–C_3_) were synthesized and characterized with the aid of elemental analysis, crystallographic, magnetic moment measurements, spectroscopic and thermogravimetric approaches. The present manuscript deals with the synthesis of Ni(II) complexes and their anticancer screening on the OVCAR-3 cell line, while antibacterial and antifungal screening by MIC. Thus, our study will provide innovative valuable insights for designing drugs for anticancer treatment.

## 2. Experimental

### 2.1. Materials and Methods

All the chemicals used were of AR grade and obtained from Sigma-Aldrich and Merck. All the solvents distilled before use as per recommended procedure. The melting points are taken into open capillaries at the Ambassador melting factor apparatus. The purity of synthesized compounds was routinely checked by thin layer chromatography (TLC) with silica gel-G (Merck). The instruments used for obtaining the spectroscopic data were IR–Thermo Fisher Scientific model Nicolet iS10; ^1^H-NMR (DMSO-*d_6_*, 400 MHZ) NMR spectrometer Bruker Avance. TMS was used as an internal standard. The electronic spectra of ligand and complexes in DMSO were recorded on Shimadzu UV-3600. The thermograms of complexes have been recorded in the temperature range 50–1000 °C using Shimadzu DTG-60 H thermal analyzer at a heating rate of 10 °C min^−1^ under an oxygen atmosphere. Mass spectra of the compounds were recorded on TOF-ES technique at 70 ev using ESI/APCI-hybrid mass spectrometer.

The reagents required to perform in vitro anticancer Sulforhodamine B (SRB) assay such as RPMI-1640, minimum essential medium (MEM), 10% fetal bovine serum, dimethyl sulfoxide (DMSO), Sulforhodamine etc. were procured from Sigma (St. Louis, MO, USA) and Hi Media Ltd., Mumbai, India. Further, a stock solution of 100 µg/mL of the all the test compounds were prepared by dissolving in 0.1% DMSO. The compounds were serially diluted and treated to the cancer cells. For cells culture, we have grown the cells and maintained in appropriate medium at pH 7.4, supplemented with 10% fetal bovine serum, glutamate (2 mM), acetic acid (1%). The cell cultures used in this study were grown in a carbon dioxide incubator (Heraeus, GmbH, Germany) at 37 °C with 90% humidity and 5% CO_2_.

### 2.2. Synthesis

#### 2.2.1. Synthesis of Ligands (HL_1_–HL_3_)

**Scheme 1:**
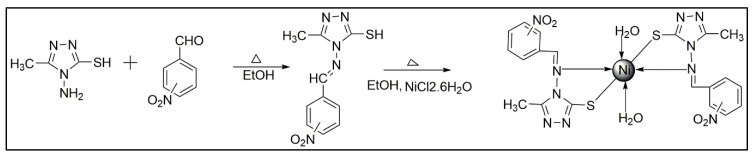
General route of synthesis of Schiff base ligand and respective Ni(II) metal complexes.

The Schiff bases (HL_1_–HL_3_) of (ortho/meta/para-nitrobenzalideneimino)-3-methyl-5-mercapto-1,2,4-triazole has been prepared by dissolving ortho/meta/para-nitrobenzaldehyde (1.511 g, 0.01 molL^−1^) and 4-Amino-5-mercapto-3-methyl-1,2,4-triazole (1.44 g, 0.01 molL^−1^) in 25 mL ethyl alcohol separately. The two ethanolic solutions were mixed thoroughly. The mixture was refluxed on a water bath for 3 h. The progress of reaction was checked by using TLC using 1:9 ethyl acetate and petroleum ether solvent mixture. On cooling, the ligands (HL_1_–HL_3_) were precipitated and are separated by filtration, washed with cold ethanol and recrystallized from ethanol and dried in vacuum over anhydrous CaCl_2_ (Scheme 1). The melting point of Schiff base HL_1_ = 224 °C, HL_2_ = 230 °C and HL_3_ = 235 °C. It is worth to note that all the geometry of the all ligands are in trans form.

#### 2.2.2. Synthesis of Metal Complexes (C_1_–C_3_)

The Schiff bases (HL_1_–HL_3_) (2.770 g, 0.01 molL^−1^) were dissolved in 25 mL ethanol and added to NiCl_2_.6H_2_O solution (1.188 g, 0.005 molL^−1^) respectively. The mixture was refluxed for 4 h. The products formed were filtered and purified thoroughly with absolute ethanol and ultimately with acetone and dried in vacuum over anhydrous CaCl_2_ (Scheme 1).

### 2.3. Biological Studies

#### 2.3.1. In Vitro Antimicrobial Studies

The antibacterial and antifungal activities of the Schiff bases (HL_1_–HL_3_) and their metal complexes (C_1_–C_3_) were tested on *Staphylococcus aureus*, *Pseudomonas aeruginosa* and *Aspergillus niger*, *Candida albicans* respectively. The method used for antibacterial activity was the Agar Well-Diffusion method [40] and for the antifungal activity Agar-Ditch method [41]. The stock solution having concentration of 1 mg mL^−1^ was prepared and was used to prepare concentrations of 0.8, 0.6, 0.4, 0.2 mg mL^−1^. The bacteria and fungi were inoculated on the surface of Nutrient agar and Sabouraud’s agar, respectively. The various concentrations of the compounds were inoculated in the wells prepared on the agar plates. The plates were incubated at room temperature for 24 h. To clarify the effect of DMSO on the biological screening, separate studies were carried out with DMSO and showed no activity against any bacteria and fungi.

#### 2.3.2. Molecular Docking Studies

Molecular docking is one of the most frequently used methods in SBDD because of its ability to predict, with a substantial degree of accuracy, the conformation of small-molecule ligands within the appropriate target binding site [42].

To find out the possible mode of action of the synthesized Schiff bases (HL_1_–HL_3_) and nickel(II) complexes (C_1_–C_3_) molecular docking calculations were carried out using biopredicta module of the V life MDS 4.4 on the crystal structure of the Human CDK 7 (PDB ID: IUA2) downloaded from Protein Data Bank (accessed on 31 August 2022: www.rcsb.org/pdb) at a resolution of 3.02. The protein structure was refined via deletion of all the hetero atoms including water molecules and the addition of the polar hydrogen atoms to get a native conformation. All other bonds were allowed to be rotatable. The structures of the synthesized metal complexes are drawn in the builder module of the V life MDS 4.4 engine. The 2D structures of the molecules were converted into the 3D structures and optimized via application of the MMFF force field. These optimized structures were further utilized for the docking analysis. The docked protein-ligand complex was further analysed via docking score of each of the complex, which is nothing but the binding energy of the complex, for every derivative 100 binding conformations were analysed to select the best conformation having the minimum docking score.

#### 2.3.3. Antiproliferative Activities against OVCAR-3 Cell Line

The cell lines were grown in RPMI 1640 medium containing 10% fetal bovine serum and 2 mM L-glutamine. For present screening experiment, cells were inoculated into 96 well microtiter plates in 90 μL at 5000 cells per well. After cell inoculation, the microtiter plates were incubated at 37 °C, 5% CO_2_, 95% air and 100% relative humidity for 24 h prior to addition of experimental drugs. Experimental drugs were dissolved in appropriate solvent to prepare stock solutions. At the time of experiment four 10-fold serial dilutions were made using complete medium. Aliquots of 10 μL of these different drug dilutions were added to the appropriate micro-titer wells already containing 90 μL of medium, resulting in the required final drug concentrations. After addition of compound, plates were incubated at standard conditions for 48 h and assay was terminated by the addition of cold TCA. Cells were fixed in situ by the gentle addition of 50 μL of cold 30% (*w*/*v*) TCA (final concentration, 10% TCA) and incubated for 60 min at 4 °C. The supernatant was discarded; the plates were washed five times with tap water and air dried. Sulforhodamine B (SRB) solution (50 μL) at 0.4% (*w*/*v*) in 1% acetic acid was added to each of the wells, and plates were incubated for 20 min at room temperature. After staining, unbound dye was recovered and the residual dye was removed by washing five times with 1% acetic acid. The plates were air dried. Bound stain was subsequently eluted with 10 mM rizma base, and the absorbance was read on an Elisa plate reader at a wavelength of 540 nm with 690 nm reference wavelength. Percent growth was calculated on a plate-by-plate basis for test wells relative to control wells.

$$\mathrm{Percentage}\;\mathrm{growth}=\frac{\mathrm{Average}\;\mathrm{absobance}\;\mathrm{of}\;\mathrm{the}\;\mathrm{cell}\;\mathrm{test}}{\mathrm{Average}\;\mathrm{absorbance}\;\mathrm{of}\;\mathrm{the}\;\mathrm{control}\;\mathrm{well}}\times 100$$

Using the six absorbance measurements [time 339 zero (Tz), control growth (C), and test growth in the presence of drug at the four concentration levels (Ti)], the percentage growth was calculated at each of the drug concentration levels. The dose response parameters were calculated for each test article. Growth inhibition of 50% (GI50) was calculated from

[(Ti − Tz)/(C − Tz)] × 100 = 50

This is the drug concentration resulting in a 50% reduction in the net protein increase (as measured by SRB staining) in control cells during the drug incubation. The drug concentration resulting in total growth inhibition (TGI) was calculated from Ti = Tz. The LC50 indicating a net loss of cells following treatment is calculated from

[(Ti − Tz)/Tz] × 100 = −50

Values were calculated for each of these three parameters if the level of activity was reached; however, if the effect was not reached or was exceeded, the values for that parameter were expressed as greater or less than the maximum or minimum concentration tested.

The results for each test agents are reported as the percentage growth of the tested cells. The compounds that reduce the growth of any one of the cell lines to 32% or less (negative numbers indicates cell kill) are passed on for evolution over a 5-log dose range. In the present screening program all the compounds possessed growth to less than 32% are regarded as active compounds. According to standard NCI protocol, maximum concentration to carry cytotoxic activity is10^−4^ M [43]. Adriamycin was served as positive control compound in the cytotoxic assay. The cell line used in the present investigation is ovary (OVCAR-3).

### 2.4. Conceptual DFT Studies

The approach for this part of the research is based on the application of Conceptual DFT for the prediction of the chemical reactivity properties of the studied molecular system [44,45,46,47,48,49]. The starting point is the calculation of their fundamental molecular structures determining the electronic densities and from these the corresponding molecular and orbital energies, mainly the HOMO and the LUMO. As usual, the many con- formers of the molecules will be predicted considering the MarvinView 17.15 software from ChemAxon [accessed on 31 August 2022: http://www.chemaxon.com]. This will be achieved with the help of the MMFF94 force field for performing Molecular Mechanics calculations [50,51,52,53,54]. Every selected conformer for each system will be subjected to a geometry optimization and frequency calculation by means of the Density Functional Tight Binding (DFTB) methodology for the obtention of suitable starting molecular structures [55]. This will be followed of a geometry reoptimization, frequency analysis and calculation of the electronic properties and the chemical reactivity descriptors of the ligands and complexed considering the MN12SX/Def2TZVP/DMSO model chemistry [56,57,58] within the context of the Kohn-Sham (KS) approach and where DMSO stands for dimethyl sulfoxide [59,60,61,62]. It is worth to remember that within Computational Chemistry, MN12SX/Def2TZVP/DMSO means using the MN12SX density functional together with the Def2TZVP basis set in the presence of DMSO as the solvent. The absence of imaginary frequencies will be checked as a guarantee that the optimized structures may be considered as minima within the energy landscape. Gaussian 16 software [63] and the SMD solvation model [64] will be considered owing to the fact that the chosen model chemistry has been previously proved in the fulfillment of the ‘Koopmans in DFT’ (KID) procedure [55,56,57,58,59,60,61,62,63,64,65,66,67,68].

## 3. Results and Discussion

**Figure 1:**
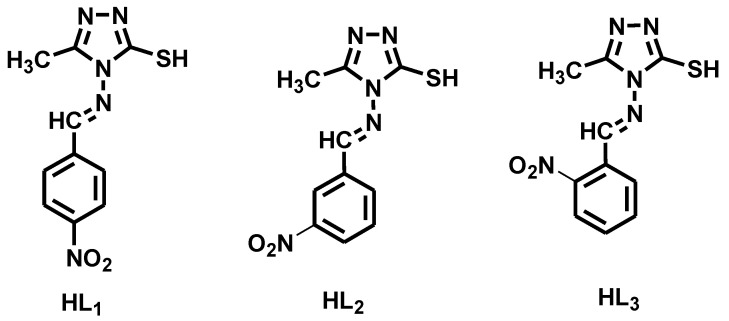
The molecular structures of triazol-based Schiff base ligands (HL_1_–HL_3_).

The synthesized Schiff bases were poorly soluble in ethanol, methanol and highly soluble in DMF and DMSO, while all the Ni(II) complexes are soluble only in DMSO. The analytical and physical data of the Schiff bases and their Ni(II) complexes are highlighted in the Table 1. The molecular structures of synthesized ligands are shown in Figure 1.

### 3.1. H-NMR and Mass Spectral Analysis

The ^1^H-NMR spectra of Schiff bases (HL_1_–HL_3_) show characteristic azomethine proton singlet sharp peak at *δ* 10.62–11.07 ppm. On the other hand, a singlet at *δ* 13.27–13.64 ppm is ascribed to -SH proton. Further, the multiplet observed at *δ* 7.27–8.29 ppm is due to typical aromatic protons. The singlet on account of -CH_3_ is observed at *δ* 2.22–2.39 ppm and singlet due to isomeric = C-CH_3_ is observed at *δ* ~2.6 ppm (Figures S1–S3). The TOF-ES mass spectra of all the compounds displayed the molecular ion peak at their respective molecular weight as depicted in Table 1.

### 3.2. FT-IR Spectral Studies

The characteristic FT-IR bands of the Schiff bases (HL_1_–HL_3_) and their Ni(II) complexes (C_1_–C_3_) are shown in Table 2. By comparing the infrared spectra of the complexes with those of free ligands one may conclude, ligand molecules exhibit thione ↔ thiol tautomerism. In the spectra of free ligands, the presence of a band at 3067–3096 cm^−1^ and 2753–2770 cm^−1^ are assigned to (N-H) and (S-H) vibrations respectively [69] (Figure S4a–c) which indicates the establishment of thione ↔ thiol tautomeric system. The deprotonation of thiol group and complexation through sulfur atom is indicated by way of the absence of a band within the range 2753–2770 cm^−1^ in the spectra of complexes. In the spectra of Schiff bases, a band due to tautomeric form of >C = S is regarded at 1114–1176 cm^−1^, in metal complexes this peak was absent. The (M-S) vibration seems in the range 332–379 cm^−1^ in the spectra of complexes [70]. The strong band at 1552–1590 cm^−1^ corresponding to azomethine group (C = N) in the spectra of free ligand was shifted to lower wave number in complexes indicating the formation of coordination bond between azomethine and metal ion [71]. This coordination is further confirmed by the presence of a band in the range 483–491 cm^−1^ in complexes assigned to (M-N) vibrations [72]. A broad band at 3311–3200 cm^−1^ indicates the presence of coordinated water molecules. The presence of a water molecule was also confirmed with the aid of thermal analysis. FT-IR of C_1_ metal complex is shown in (Figure S4d).

### 3.3. Electronic and Magnetic Moment Studies

The electronic spectra of Schiff bases (HL_1_–HL_3_) and their Ni(II) complexes (C_1_–C_3_) have been measured at room temperature by using DMSO as a solvent (Figure S5a–f). The electronic spectra of Schiff bases suggest a band between 335 and 352 nm which may be assigned to n→π* transition of azomethine group. This transition in metal complexes was shifted to lower frequency indicating that imine nitrogen is involved in the coordination of metal ion [73].

All the Ni(II) complexes exhibits three absorption bands at 990–1012 nm (γ_1_), 580–620 nm (γ_2_) and ~400 nm (γ_3_) [64,65,66]. These peaks assigned to ^3^A_2g_ (F) →^3^T_2g_ (F) (γ_1_), ^3^A_2g_ (F) → ^3^T_1g_ (F) (γ_2_), and ^3^A_2g_ (F) → ^3^T_1g_ (P) (γ_3_) transitions respectively which indicates the distorted octahedral geometry for Ni(II) ion. The measured magnetic moment for the Ni(II) complexes at 3.23–3.45 BM, recommended distorted octahedral geometry [74]. The electronic spectral data and magnetic moment values of Ni(II) complexes are summarized in Table 3.

### 3.4. Thermal Analysis

In thermal analysis, it is observed that, the Ni(II) complexes decomposes in three stages (Table 4). The first stage is between 120 and 190 °C consequences in the mass loss of coordinated water molecules of hydration. Then the anhydrous complexes decompose in a major stage consisting of two overlapping steps. In the first step, the organic moiety started decomposing, leaving metal-triazole at 180–455 °C. In the temperature range of 400–560 °C, all the triazole parts get decomposed. The decomposition of the complexes ends with nickel oxide formation above 550 °C. These observations are matched with existing literature [75]. The thermogram of [Ni(L_1_)_2_(H_2_O)_2_] is represented in Figure S6.

### 3.5. X-ray Diffraction Analysis

The X-ray powder diffraction method is broadly used as an experimental technique to purpose of crystal structure. It is more suitable for identification and determination of crystal structure of high symmetry. The X-ray diffraction of compounds was carried out in the range 5–100 °C at wavelength of 1.54060 Å. The diffractogram and associated data describe the 2θ value for each peak, relative intensity and inter planar spacing. The diffractogram of HL_1_ had nine reflections between 20–60° with maximum at 2θ = 26.6576° corresponding to d value 3.34404Å. The diffractogram of HL_2_ shows eleven reflections with maximum at 2θ = 11.3588° corresponding to d value 7.92995Å. The diffractogram of HL_3_ had eleven reflections with maxima at 2θ = 11.3588° corresponding to d value 7.79024Å. The diffractogram of C_3_ had eleven reflections with maxima at 2θ = 7.4380° corresponding to d value 11.88565Å. The X-ray diffraction pattern of these compounds with respect to major peaks having relative intensity 100% has been indexed by using computer programme. The above indexing method also yields Miller indices (*hkl*), unit cell parameters and unit cell volume. The X-ray diffraction spectra of all compounds are shown in Figure S7a–d and their d values, FWHM and relative intensities are given in Tables S1–S4. Also X-ray parameters are listed in Table 5. In concurrence with cell parameters, the conditions such as a ≠ b ≠ c and α = β = γ = 90° required for samples to be orthorhombic were tested and found to be satisfactory. Hence, it can be concluded that all the compounds have orthorhombic crystal system [76]. The experimental density values for all the ligands and their metal complexes were determined by using standard specific gravity method [77,78] and found to be 0.7512, 0.2016, 0.61 and 0.47 g/cc for HL_1_, HL_2_, HL_3_ and C_3_, respectively. By using experimental density values, molecular weight, Avogadro’s number, volume of the unit cell, the number of molecules per unit cell were calculated using the equation ρ = ηM/NV and was found to be one, one, two, one for HL_1_, HL_2_, HL_3_ and C_3_, respectively. With these values theoretical densities were computed and found to be 0.8239, 0.2265, 0.65 and 0.52 g/cc for the respective compounds. The comparison of experimental and theoretical density values shows good agreement within the limits of experimental error [79,80].

To calculate pore fraction ‘P’ the experimental and theoretical densities of sample are needed. The pore fraction ‘P’ is determined by relation P = 1 − (Experimental density/Theoretical density). By substituting density values, ‘P’ was calculated. The *p* values are observed 0.0853, 0.1099, 0.0599 and 0.0961 for the respective compounds. In fact this approximation is not sufficient for exact picture of pore fraction parameter, but this value is very important in obtaining information about inhomogeneity of sample. Other method to study inhomogeneity of sample is related with the average particle size. The average particle size (Crystallite size) was calculated using line broadening with Debye-Scherrer equation. D = 0.9λ/βCosθ. The crystallite size is observed 337, 169, 337 and 235 Å for the respective compounds. Micro strain is calculated as Microstrain = βCosθ/4 and found to be 1.2649 × 10^−3^, 219 × 10^−3^, 1.024 × 10^−3^ and 1.376 × 10^−3^ for the respective compounds. The space group of all compounds was confirmed by referring to earlier reported reference [81,82,83,84,85].

### 3.6. Conceptual DFT Report

The Kohn-Sham (KS) methodology includes the determination of the molecular energy, the electronic density and the orbital energies of a given system, in particular, the frontier orbitals HOMO (Highest Occupied Molecular Orbital) and LUMO (Lowest Unoccupied Molecular Orbital) which are intrinsically related to the chemical reactivity of the molecules [86,87,88,89]. The goodness of a given density functional can be determined through a comparison of the results that it renders with the experimental values or with the results that can be obtained by means of high-level calculations. However, the lack of experimental results for the molecular systems under study or the large size of the molecules sometimes ren- der this comparison as computationally impractical. A methodology called KID has been developed by some of the authors [90,91,92,93], for the validation of a given density functional in terms of its internal coherence. It has been shown that within the Generalized Kohn-Sham (GKS) version of DFT, there are some relations between the KID methodology and the Ionization Energy Theorem, which is a corollary of Janak theorem [94,95,96,97]. This done by connecting *E_H_* to -I, *E_L_* to -A, and a combination of both orbital energies through the formulas *J_I_* = *E_H_* + *E_gs_*(N − 1) − *E_gs_*(N), *J_A_* = *E_L_* + *E_gs_*(N) − *E_gs_*(N + 1), and *J_HL_* = *J* + *J*, being *E*_L_ and *E*_H_ the HOMO and LUMO energies related to the molecular systems considered in this research. An additional KID descriptor ∆SL amounting to the difference in energies between the SOMO (equivalent to the HOMO of the radical anion) and the LUMO of the neutral system has been designed to help in the verification of the accuracy of this methodology [75,76,77,78]. The results for these calculations are presented in Table 6.

The KID methodology is simply a calculation of the difference between how I and A are estimated: as energy differences or resorting to the HOMO and LUMO energy orbitals. This allows us to check if the considered density functional (and its associated model chemistry) verifies the agreement with the Janak and Ionization Energy theorems. Thus, the values displayed on Table 6 have been calculated using the calculated electronic energies of the neutral, cation and anion, as well as the HOMO and LUMO orbital energies with the presented KID formulas. For a perfect agreement, the values of the KID descriptors must be equal to zero. However, the low values of them make us confidents that the used methodology is adequate for the purpose of this research. Moreover, we have shown earlier that in the presence of water and DMSO as solvents, the MN12SX functional be- haves better in the verification of the Janak and Ionization Energy theorems than other usual long-range corrected density functionals, which in turn, are better when working in the gas phase or absence of solvent [88]. Although this agreement is not perfect, the results make us confidents about the goodness of the energies related to the frontier orbitals so as to make possible to calculate the Conceptual DFT descriptors directly from them.

As it is well known, the energy related to the HOMO- LUMO transitions represents a good approximation to that corresponding to the maximum wavelength absorption in a UV-Vis spectrum. As can be seen from the experimental spectra presented in the Supplementary Materials, this absorption takes place at larger wavelengths for the complexes than for the ligands. Thus, their HOMO- LUMO gaps will be narrower. Indeed, for a quantitative agreement TD-DFT calculations will be needed, but our obtained HOMO-LUMO gaps represents a qualitative pictorial example of this effect.

This methodology is convenient when thinking of quantitative qualities related with Conceptual DFT descriptors [89,90]. The definitions for the global reactivity descriptors are [34,35,36,37,79,80]: Electronegativity as *χ ≈* ^1^ (*E_L_* + *E_H_*), Global Hardness as *η ≈* (*E_L_ − E_H_*), Electrophilicity as *ω* *≈* (*E_L_* + *E_H_*)^2^*/*4(*E_L_ − E_H_*), Electrodonating Power as *ω^−^ ≈* (3*E_H_* + *E_L_*)^2^*/*16*η*, Electroaccepting Power as *ω*^+^ *≈* (*E_H_* + 3*E_L_*)^2^*/*16*η* and Net Electrophilicity as ∆*ω^±^* = *ω*^+^ *−* (*−ω^−^*) = *ω*^+^ + *ω^−^*. These global reactivity descriptors that arise from Conceptual DFT [79,80], has been complemented by a Nucleophilicity Index N [81,82,83,84,85] that takes into account the value of the HOMO energy obtained by means of the KS scheme using an arbitrary shift of the origin with tetracyanoethylene (TCE) as a reference. The results for the determination of the Conceptual DFT reactivity descriptors for the ligands and their Ni(II) complexes are displayed in Table 7.

It can be seen from Table 7 that the electrophilicity *ω* is lower for the ligands than for the complexes and the same trend is observed for the nucleophilicity N. In- deed, this has to be with the presence of the Ni(II) ion in the complexes although this effect also depends on the different positions of the -NO_2_ group within the ligands. The same conclusions can be extracted from the *ω^−^*, *ω*^+^ and ∆*ω^±^* results, being also the *ω^−^* values greater in the cases than the *ω*^+^, which gives an indication of their reactivity behavior.

### 3.7. In Vitro Antimicrobial and Antifungal Activities

All the Schiff bases (HL_1_–HL_3_) and Ni(II) complexes (C_1_–C_3_) are inactive against *Aspergillus niger* (Table 8). The ligand is weakly active against *Pseudomonas aeruginosa and Candida albicans* while moderate to highly active against *Staphylococcus aureus*. All the Ni(II) complexes are moderate to highly active against bacteria and moderately active against *Candida albicans*. The results were compared with standard drugs like Gentamycine and Streptomycin. The compounds are less potent as compare to standard drugs.

**Figure 2:**
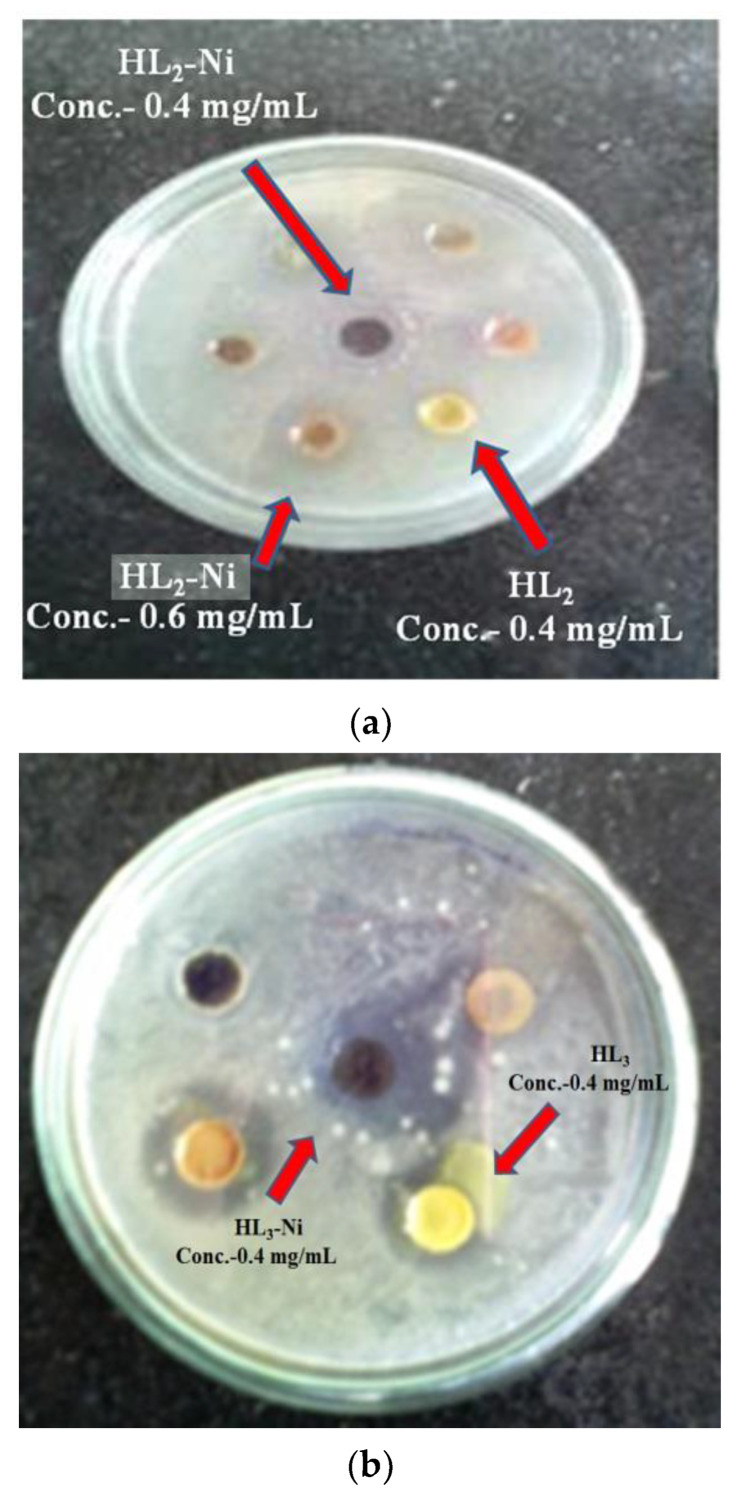
(**a**) Zone of inhibition of HL_2_ and HL_2_-Ni for *Pseudomonas aeruginosa;* (**b**) Zone of inhibition of HL_3_ and HL_3_ -Ni for *Staphylococcus aureus*.

Similar procedure was repeated for antifungal activities; the Malt extract-Glucose-Yeast extract-Peptone (MGYP) agar plates were prepared using submerged inoculation using fungal strain *Candida albicans* (NCIM 3466). The agar plates were incubated at 27 °C for 48 to 72 h. After incubation plates were examined for zone of inhibition around wells as shown in Figure 2.

### 3.8. Molecular Docking Studies

Following the development of the first algorithms in the 1980s, molecular docking is becoming an essential tool in drug discovery [32]. For example, the investigations involving crucial molecular events, including ligand binding modes and the corresponding intermolecular interactions that stabilize the ligand-receptor complex, can be conveniently performed [33]. Furthermore, molecular docking algorithms executes quantitative prediction of binding energetics, providing rankings of docked compounds based on the binding affinity of ligand-receptor complexes.

In order to understand the possible mode of action of the synthesized metal complex, the molecular docking analysis was carried out using crystal structure of the Human CDK7. Docking analysis revealed that HL_1_ (GI50 > 100 μM) is interacting with the selected protein target with formation of the only one hydrogen bond interaction with GLN22 and hydrophobic interaction with the amino acids like LYS41, VAL36 with total docking score of −40.52 k Cal mol^−1^. C_1_ complex (GI50 = 66.7 μM) was found to have docking score of -84.53 k Cal mol^−1^ and interacted via formation of one hydrogen bond interaction with ASN142, charge interaction with GLU99, ASP97 and hydrophobic interactions with GLY21 and GLU99. HL_2_ (GI50 > 100 μM) was found to be showing docking score of −47.89 k Cal mol^−1^ and interacted via formation of two hydrogen bond interactions with PHE23, SER97, aromatic interaction with TRP43, PHE51, PHE122, van der Waals interactions with TYR18, PHE40, TRP43, SER44 etc. and hydrophobic interaction with LEU144, GLU121, PHE122, ALA125. C_2_ (GI50 = 67.7 μM) complex interacted via formation of one hydrogen bond interaction with ASN142, charge interaction with GLU99 and hydrophobic interaction with LYS103, ASN144 with total docking score of −96.35 k Cal mol^−1^. HL_3_ (GI50 > 100μM) showed docking score of -47.89 k Cal mol^−1^ and interacted via formation of hydrogen bond interaction with PHE23 and hydrophobic interaction with LEU144, LYS41. The C_3_ complex (GI50 > 100 μM) was found to have docking score of −88.76 k Cal mol^−1^ and showed one hydrogen bond interaction with LYS139, charge interaction with ASP97 and hydrophobic interaction with LYS139, VAL100. Docking analysis indicated the developed ligands are having ability to bind with the Human CDK7 which can be possible mode of the action for anticancer potential.

**Figure 3:**
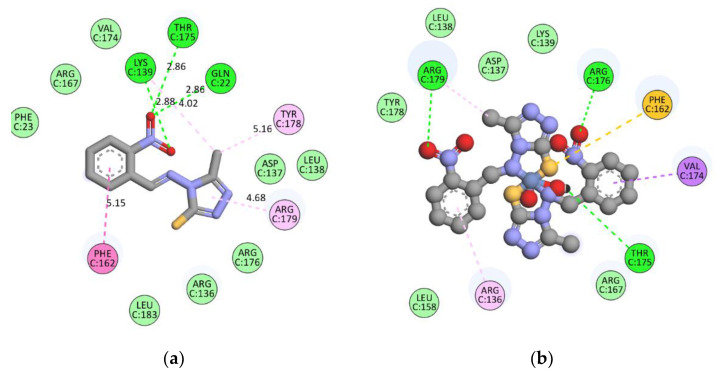
Interactions of (**a**) HL_1_ and (**b**) C_1_ with amino acids (LYS41 and VAL36).

The binding energy and critical interactions of all Schiff bases (HL_1_–HL_3_) and their Ni(II) complexes (C_1_–C_3_) are exhibited in the Table 9. The interactions of HL_1_ and C_1_ with amino acid are exhibited in Figure 3.

### 3.9. Effect of ligands (HL_1_–HL_3_) and its complexes (C_1_–C_3_) on antiproliferative activity

**Figure 4:**
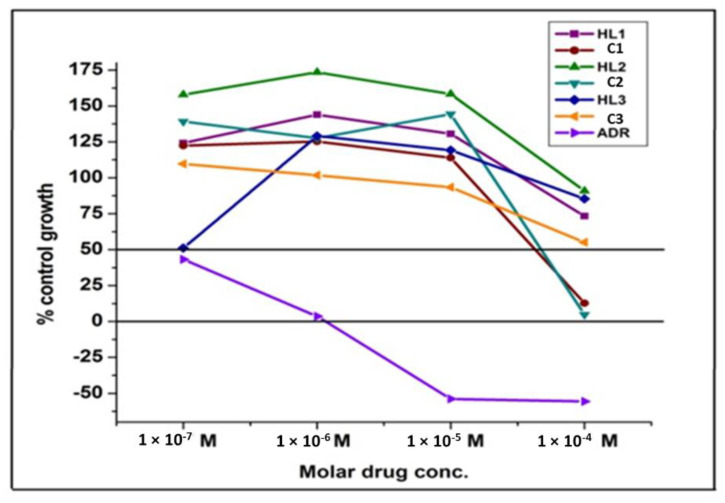
Growth curve for human ovarian cancer cell line OVCAR-3 for Schiff bases and their Ni(II) complexes.

The growth curve of human ovarian cancer cell line OVCAR-3 for Schiff bases (HL_1_–HL_3_) and their Ni(II) complexes (C_1_–C_3_) is represented in Figure 4. All the synthesized compounds were screened for their anticancer activity against OVCAR-3 (ovary) cell line. The anticancer activity was measured in vitro for the newly synthesized Schiff bases (HL_1_–HL_3_) and Ni(II) complexes (C_1_–C_3_) using the Sulforhodamine B stain (SRB) assay method [97,98,99]. In the current protocol, each cell line is inoculated on a pre-incubated microtiter plate. The test agents are added at a single concentration and the culture is incubated for 48 h. The endpoint of determinations is made with Sulforhodamine B, a protein-binding dye. The results for each test agent are reported as the percentage growth of the tested cells. The average values for % control growth for the cell line OVCAR-3 are listed in Table 10.

**Figure 5:**
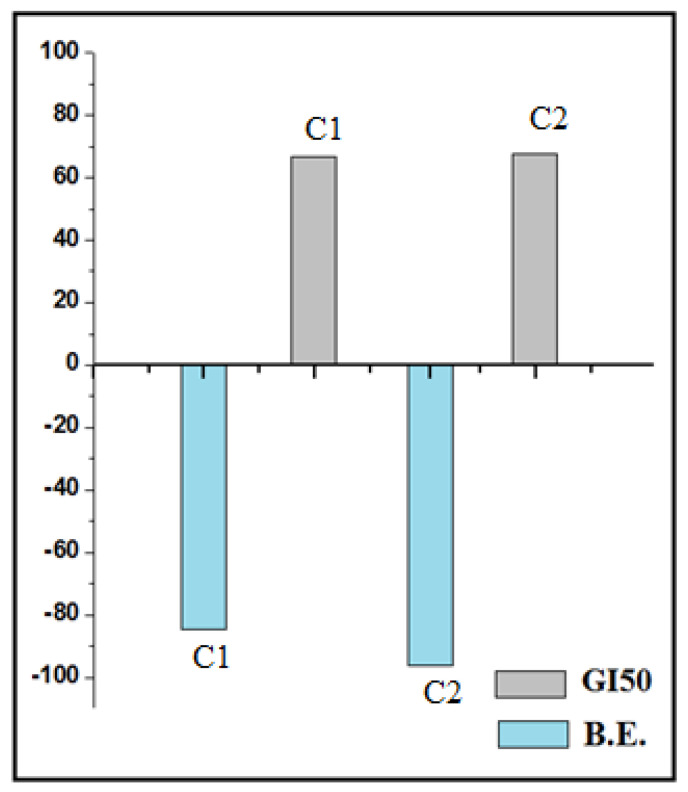
Correlation plot of binding energy and GI50 for C_1_ and C_2_.

The results concern with average values for % control growth suggests that all the Schiff bases are inactive against ovarian cancer cell line OVCAR-3 at all concentrations but C_1_ and C_2_ are active at 10^−4^ mol L^−1^ concentration. C_2_ is more active than C_1_ at 10^−4^ mol L^−1^ concentration. The C_3_ does not show any activity. Based on GI50 value all the Schiff bases are inactive while C_1_ (GI50 = 66.7 × 10^−6^mol L^−1^) and C_2_ (GI50 = 67.7 × 10^−6^mol L^−1^) are moderately active against ovarian cancer cell line OVCAR-3. C_3_ is inactive towards cancer cell lines OVCAR-3. The correlation plot of binding energy and GI50 for active compounds (C_1_ and C_2_) is represented in Figure 5.

### 3.10. Structure Activity Relationship (SAR Studies)

In general, synthesized Ni(II) complexes are found to be active against OVCAR-3 cell lines at a molar dose of 10^−4^ molL^−1^ which indicates their anticancer potential. The SAR analyses of the synthesized complexes are indicated in the following:

1, 2, 4-Triazoles with methyl substituent were moderately active against cancer cell lines. The Ni(II) complexes of HL_1_ and HL_2_ are showed GI50 value 66.7 and 67.7 μM respectively, which indicates the substitution of the good electron-withdrawing group like -NO_2_ sufficiently affecting the polarity of the molecules.

The position of the -NO_2_ group is also an important parameter in the biological activity of these derivatives, 3 and 4 substituted derivatives are more potent than the corresponding 2 substituted ones. This might be due to the loss of favourable conformation in ortho-substituted derivatives.

## 4. Conclusions

In summary, the synthesized Schiff bases act as a bidentate ligand and coordinated to the Ni(II) ion through imine nitrogen and sulphur of the thiol group. The binding of ligand to a metal ion is confirmed by elemental analysis, spectral studies (UV-Visible, IR, ^1^H-NMR), TGA and magnetic moment measurement. The Ni(II) complexes are found to exhibit octahedral geometry. All the Schiff bases (HL_1_–HL_3_) and their complexes are inactive against *Aspergillus niger* but metal complexes are more effective against *Staphylococcus aureus, Pseudomonas aeruginosa* and *Candida albicans*. The anticancer results revealed that Ni(II) complexes exhibit significant anticancer activity than their corresponding ligands.

## Figures and Tables

**Table 1:** Physical and analytical data of ligands and metal complexes.

Sl. No.	Name of Comp.	Mol. Formula	Mol. Wt	Color	Elemental Analysis			
					C% Found (Calc.)	H% Found (Calc.)	N% Found (Calc.)	M% Found (Calc.)
1	HL_1_	C_10_H_9_N_5_SO_2_	263	Pale yellow	45.20 (45.63)	3.52 (3.42)	26.48 (26.61)	-
2	C_1_	C_20_H_20_N_10_S_2_O_6_Ni [Ni(L_1_)_2_2H_2_O]	618.69	green	38.71 (38.78)	3.30 (3.23)	22.58 (22.62)	9.59 (9.52)
3	HL_2_	C_10_H_9_N_5_SO_2_	263	Yellow	45.78 (45.63)	3.45 (3.42)	26.70 (26.61)	-
4	C_2_	C_20_H_20_N_10_S_2_O_6_Ni [Ni(L_2_)_2_2H_2_O]	618.69	green	38.66 (38.78)	3.31 (3.23)	22.71 (22.62)	9.61 (9.52)
5	HL_3_	C_10_H_9_N_5_SO_2_	263	Dark yellow	45.52 (45.63)	3.55 (3.42)	26.65 (26.61)	-
6	C_3_	C_20_H_20_N_10_S_2_O_6_Ni [Ni(L_3_)_2_2H_2_O]	618.69	Yellow green	38.70 (38.78)	3.28 (3.23)	22.49 (22.62)	9.40 (9.52)

**Table 2:** FT-IR spectral data of ligands and metal complexes (cm^−1^).

Sl. No.	Compd.	ν_C = N_	ν_SH_	ν_C = S_	ν_N-H_	ν_H__2O_	ν_M-S_	ν_M-N_
1	HL_1_	1590	2760	1114	3067	-	-	-
2	C_1_	1525	-	-	-	3206	379	483
3	HL_2_	1589	2753	1176	3072	-	-	-
4	C_2_	1568	-	-	-	3311	332	491
5	HL_3_	1579	2770	1170	3096	-	-	-
6	C_3_	1552	-	-	-	3200	379	483

**Table 3:** Electronic and magnetic moment studies of complexes.

Complex	γ1 (nm)	γ2 (nm)	γ3 (nm)	µ Eff. (BM)
C1	1011	620	400	3.45
C2	990	580	400	3.23
C3	1012	616	401	3.34

**Table 4:** Stepwise thermal degradation of metal complexes (C_1_–C_3_).

Compound	Stages	Temp. (°C)	TG Mass %		Assignment
			Calc.	Found	
C_1_	1 2 3	50–180 180–400 400–550 >550	5.81 48.14 36.51 12.10	6.92 46.11 34.09 12.94	-2.H_2_O (water molecules) -C_14_H_10_N_4_O_4_ (organic moiety) -C_6_H_6_N_6_S_2_ (triazole ring) -NiO (residue)
C_2_	1 2 3	50–190 190–405 405–555 >555	5.81 48.14 36.51 12.10	6.90 46.16 34.11 12.90	-2.H_2_O (water molecules) -C_14_H_10_N_4_O_4_ (organic moiety) -C_6_H_6_N_6_S_2_ (triazole ring) -NiO (residue)
C_3_	1 2 3	50–165 165–435 435–560 >560	5.81 48.14 36.51 12.10	6.71 47.07 34.70 12.90	-2.H_2_O (water molecules) -C_14_H_10_N_4_O_4_ (organic moiety) -C_6_H_6_N_6_S_2_ (triazole ring) -NiO (residue)

**Table 5:** X-ray parameters of HL_1_, HL_2_, HL_3_ and C_3_.

Sl. No.	Parameter	HL_1_	HL_2_	HL_3_	C_3_
1	Structure	Orthorhombic	Orthorhombic	Orthorhombic	Orthorhombic
2	Space group	*I mam*	*I mam*	*I mam*	*I mam*
3	Symmetry of lattice	Primitive	Primitive	Body centered	Primitive
4	Bond angle	α = β = γ = 90°	α = β = γ = 90°	α = β = γ = 90°	α = β = γ = 90°
5	Lattice parameters (Å)	a = 16.61 b = 08.16 c = 03.91	a = 14.40 b = 18.52 c = 07.22	a = 10.83 b = 16.08 c = 07.93	a = 11.89 b = 13.24 c = 12.63
6	Volume of the cell (V) (Å^3^)	529.96	1927.23	1343.12	1987.79
7	Density (g/cc) Theoretical Experimental	0.8239 0.7512	0.2265 0.2016	0.65 0.61	0.52 0.47
8	No. of molecules/unit cell (η)	1	1	2	1
9	Pore fraction	0.0853	0.1099	0.0599	0.0961
10	Crystallite size (Å)	337	169	337	235
11	Micro strain (×10^−3^)	1.2649	219	1.024	1.376

**Table 6:** Frontier Orbital Energies, HOMO-LUMO Gap and the KID Descriptors of the Ligand Descriptors and their Ni(II) Complexes.

Compound	HOMO	LUMO	SOMO	H-L Gap	J*_I_*	J*_A_*	J*_HL_*	∆SL
HL_1_	−6.31	−3.12	−2.69	3.19	0.25	0.23	0.34	0.43
HL_2_	−6.28	−2.89	−2.56	3.39	0.24	0.19	0.31	0.33
HL_3_	−6.30	−3.05	−2.73	3.25	0.24	0.17	0.21	0.31
C_1_	−5.17	−3.19	−2.87	1.98	0.45	0.18	0.48	0.32
C_2_	−5.66	−3.03	−2.83	2.63	0.13	0.11	0.17	0.21
C_3_	−5.77	−3.04	−2.85	2.73	0.14	0.10	0.17	0.19

**Table 7:** Global Reactivity Descriptors of the Ligands and their Ni(II) Complexes.

Compound	*X*	η	ω	S	N	ω^−^	ω^+^	Δ ω^±^
HL_1_	4.72	3.19	3.47	0.31	2.48	9.53	4.81	14.35
HL_2_	4.59	3.39	3.10	0.29	2.51	8.70	4.12	12.82
HL_3_	4.67	3.25	3.36	0.31	2.50	9.25	4.58	13.84
C_1_	4.18	1.98	4.41	0.51	3.63	11.03	6.85	17.88
C_2_	4.35	2.63	3.60	0.38	3.13	9.53	5.18	14.71
C_3_	4.40	2.73	3.55	0.37	3.02	9.47	5.06	14.53

**Table 8:** Antimicrobial activity of Schiff bases and their Ni(II) complexes.

Sr. No.	Compd.	Conc.mg mL^−1^	*Pseudomonas* *Aeruginosa*	*Staphylococcus* *aureus*	*Aspergillus* *niger*	*Candida* *albicans*
1	HL_1_	0.2 0.4 0.6 0.8 1.0	+++ +++ +++ +++ +++	+++ +++ +++ +++ +++	- - - - -	- - - - -
2	HL_1_-Ni	0.2 0.4 0.6 0.8 1.0	+++ +++ +++ +++ +++	+++ +++ +++ +++ +++	- - - - -	- + ++ +++ +++
3	HL_2_	0.2 0.4 0.6 0.8 1.0	- ++ ++ - ++	++ +++ +++ +++ ++	- - - - -	++ ++ ++ ++ +++
4	HL_2_-Ni	0.2 0.4 0.6 0.8 1.0	- - ++ - +++	++ ++ +++ +++ +++	- - - - -	- - - ++ -
5	HL_3_	0.2 0.4 0.6 0.8 1.0	+ ++ ++ +++ +++	++ ++ +++ +++ +++	- - - - -	- - - +++ +++
6	HL_3_-Ni	0.2 0.4 0.6 0.8 1.0	- - - ++ ++	++ +++ +++ +++ +++	- - - - -	- - - + +

Less than 5 mm = Inactive (-), 6–10 mm = Weakly active (+), 11–14 mm = Moderately. Active (++), 15 mm and above = Highly active (+++).

**Table 9:** Binding Interaction of Schiff bases and their Ni(II) complexes.

Sr. No.	Molecule Code	B. E. (k Cal mol^−1^)	Interaction with Amino Acid		
			H Bond	Charge	Hydrophobic
1	HL_1_	−40.52	GLN22	---	LYS41, VAL36
2	C_1_	−84.53	ASN142	GLU99, ASP97	GLY21, GLU99
3	HL_2_	−47.89	PHE23, SER97	π stackang-TRP43, PHE51, PHE122 VDW*- TYR18, PHE40, TRP43, SER44 etc.	LEU144, GLU121, PHE122, ALA125
4	C_2_	−96.35	ASN142	GLU99,	LYS103, ASN144
5	HL_3_	−47.89	PHE23	---	LEU144, LYS41
6	C_3_	−88.76	LYS139	ASP97	LYS139, VAL100

* VDW-van der Waals forces.

**Table 10:** Preliminary in vitro anticancer screening data of Schiff bases (HL_1_–HL_3_) and their complexes (C_1_–C_3_).

Conc. (Molar)	Average Values for % Control Growth						
	HL_1_	C_1_	HL_2_	C_2_	HL_3_	C_3_	Adriamycin
10^−7^	124.1	122.4	157.9	139.2	51.1	109.7	43.2
10^−6^	144	125.3	173.4	127.6	129.1	101.8	3.6
10^−5^	130.6	113.9	158.3	144.4	119.2	93.5	−53.9
10^−4^	73.4	12.7	90.9	4.8	85.4	55.1	−55.6

## Data Availability

Not applicable.

## Supplementary Materials

The following supporting information can be downloaded at: https://www.mdpi.com/article/10.3390/molecules27196548/s1, Figures S1-S3: 1H-NMR of ligands; Figure S4: IR spectra of ligand and complex; Figure S5: UV-Vis of ligand and complexes; Figure S6: TGA/DTA thermogram; Figure S7: X-ray diffractograms of ligands and complex; Table S1: XRD data of ligand and complex.

## Sample Availability

Samples of the compounds are available from the authors upon requisition.
