# Supplementary material for: In Vitro Anticancer Screening, Molecular Docking and Antimicrobial Studies of Triazole-Based Nickel(II) Metal Complexes
Source: Molecules. 2022 Oct 3;27(19):6548. doi: 10.3390/molecules27196548 (PMC9570899; doi:10.3390/molecules27196548)
Supplement: Supplementary file 1 [file molecules-27-06548-s001.zip › molecules-1932824-supplementary.pdf]

Supporting Information

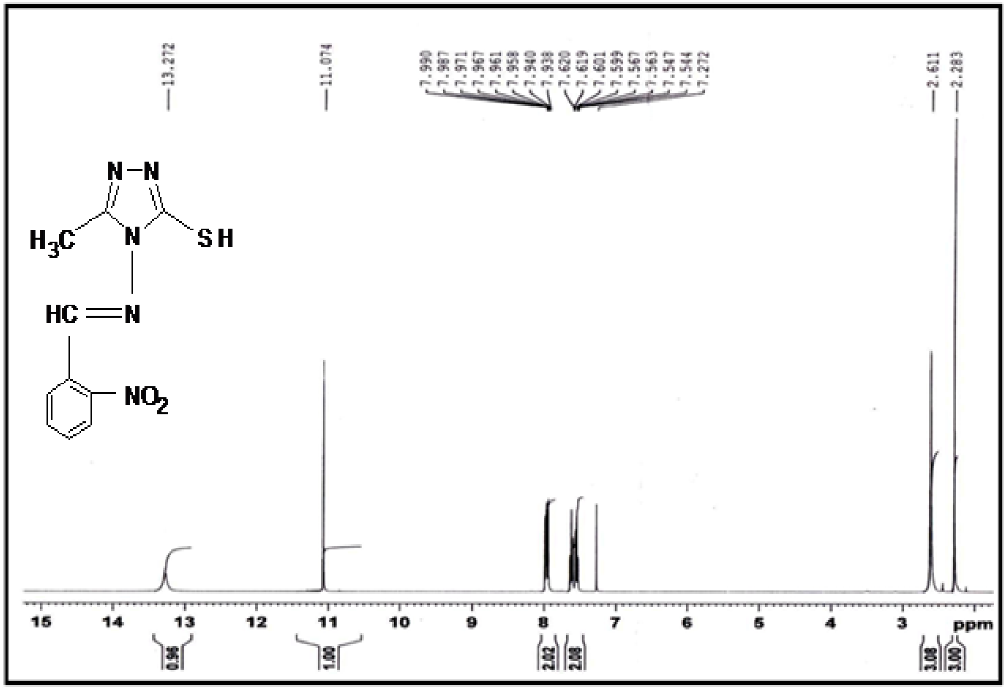

Figure S1: ^{1}H-NMR of HL_1: 4-(2′-Nitrobenzelideneimino)-3-methyl-5-mercapto-1,2,4-Triazole.

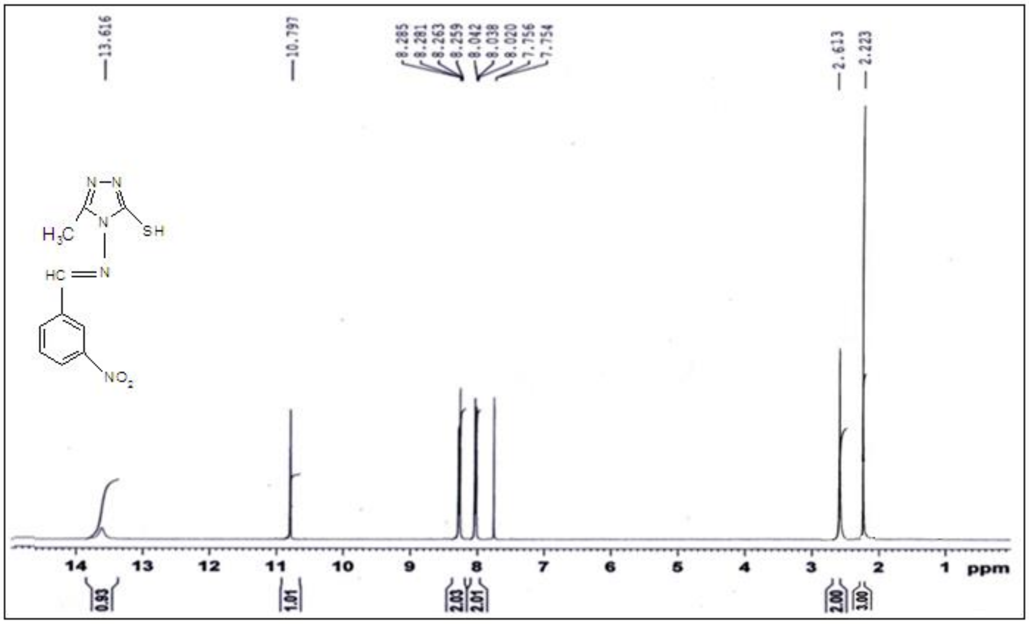

Figure S2: ^{1}HNMR spectrum of HL_2 **(** 4-(3′-Nitrobenzelideneimino)-3-methyl-5-mercapto-1,2,4-triazole).

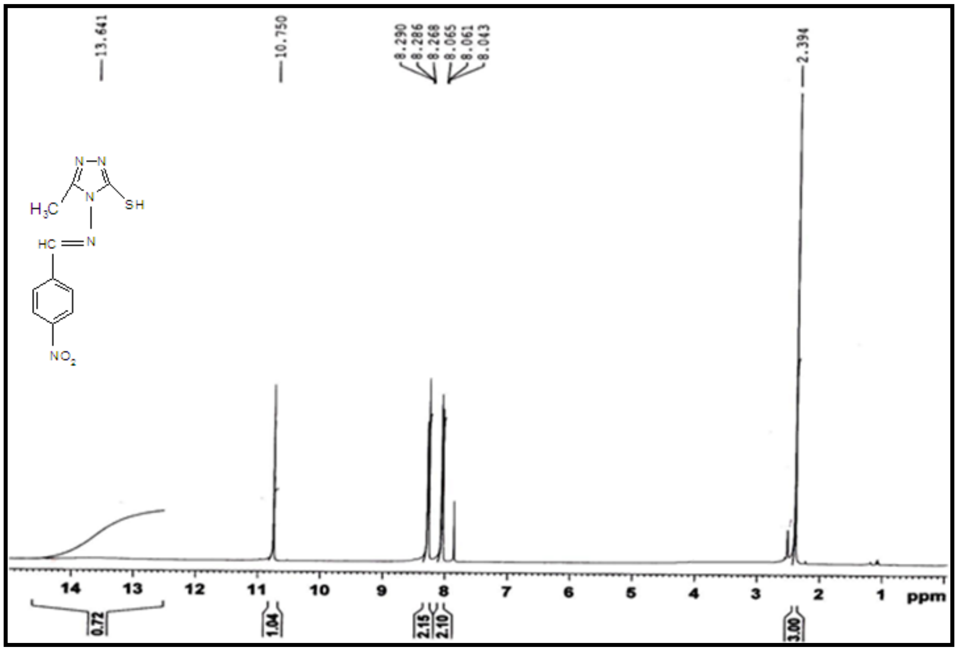

Figure S3: ^{1}HNMR spectrum of HL_3 (4-(4′-Nitrobenzelideneimino)-3-methyl-5-mercapto-1,2,4-triazole).

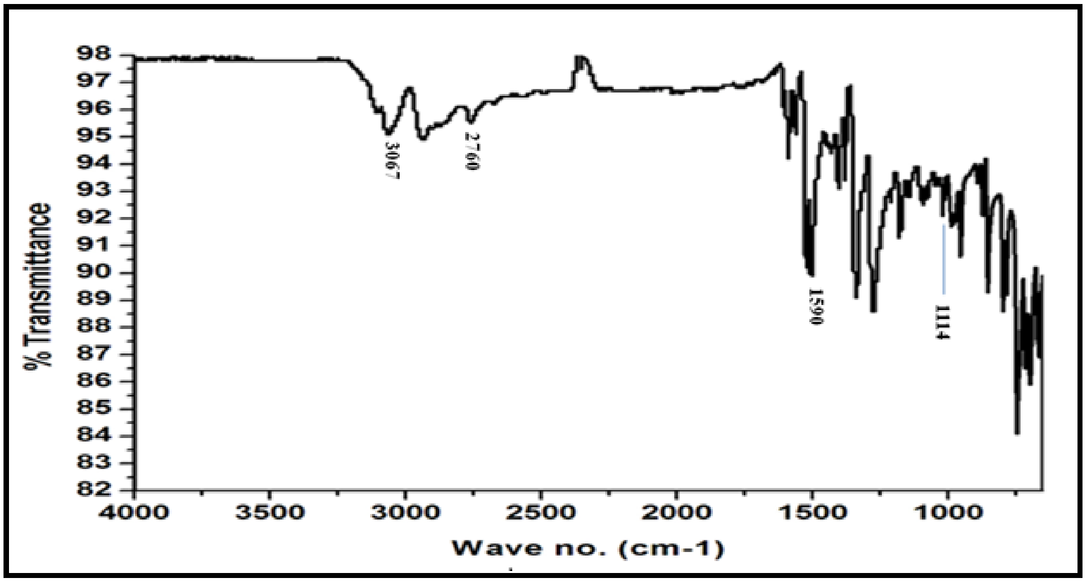
% Transmittance
98
97
96
95
94
93
92
91
90
89
88
87
86
85
84
83
82
4000
3500
3000
2500
2000
1500
1000
Wave no. (cm-1)
3067
2760
1590
1114

(a)

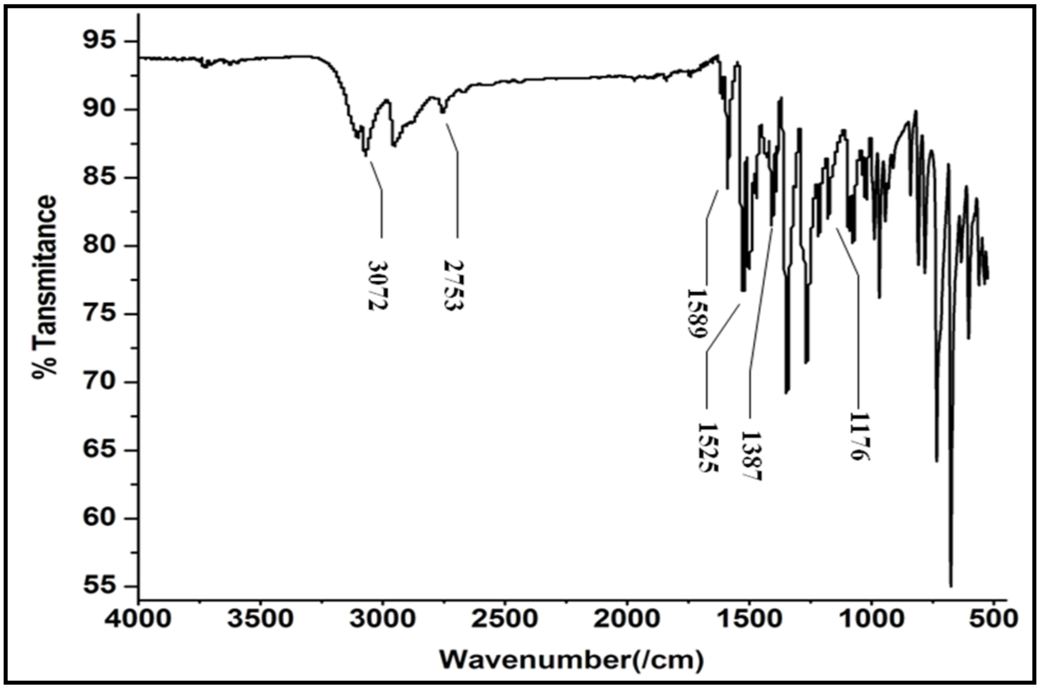
% Tansmittance
95
90
85
80
75
70
65
60
55
4000
3500
3000
2500
2000
1500
1000
500
Wavenumber(/cm)
3072
2753
1589
1525
1387
1176

(b)

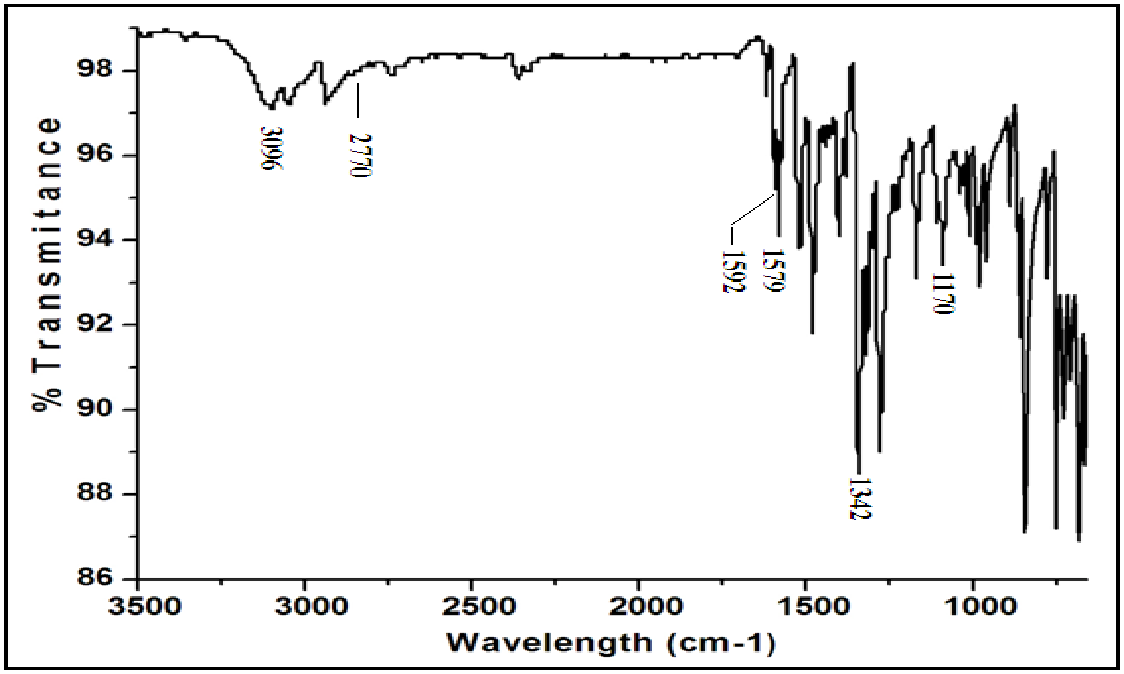

(c)

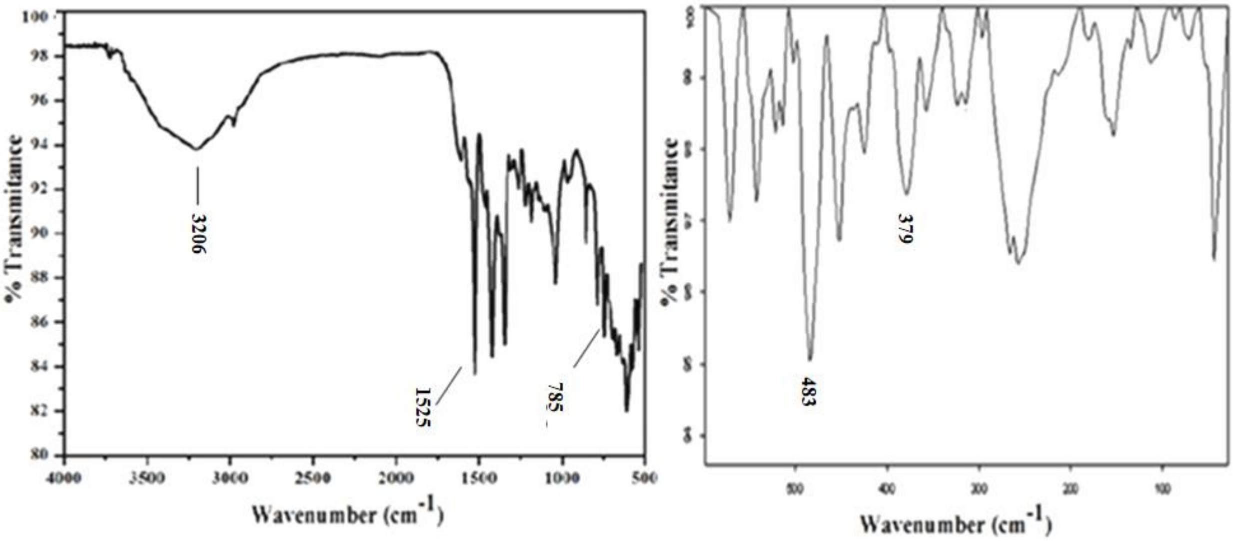

(d)

Figure S4: FT-IR spectra of (a) HL_1, (b) HL_2, (c) HL_3 and (d) complex $\mathbf{C_1}$.

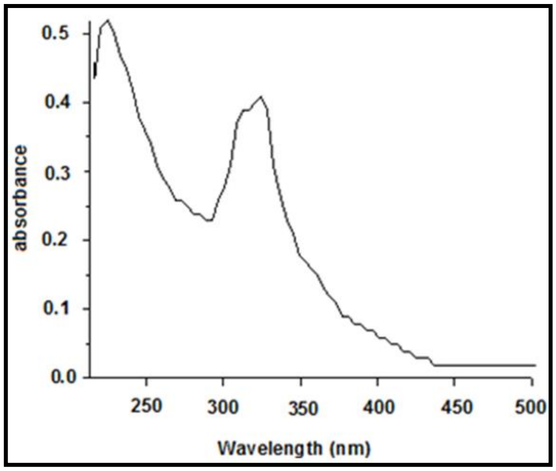
absorbance
0.5
0.4
0.3
0.2
0.1
0.0
250
300
350
400
450
500
Wavelength (nm)

(a)

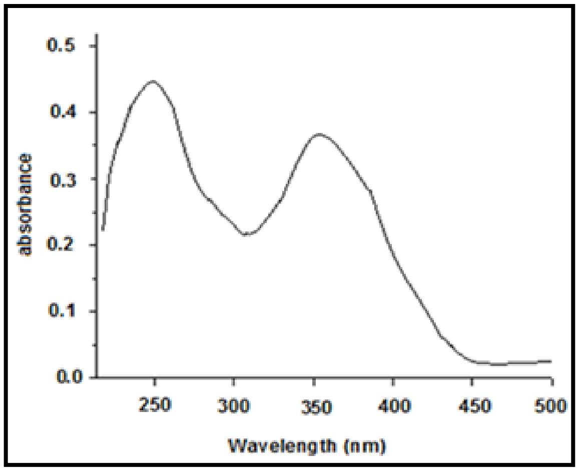
absorbance
0.5
0.4
0.3
0.2
0.1
0.0
250
300
350
400
450
500
Wavelength (nm)

(b)

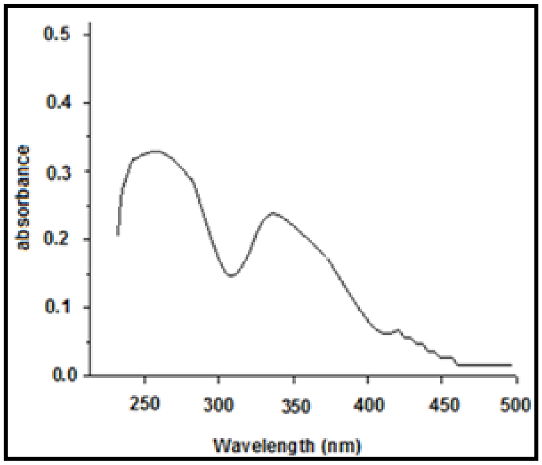
absorbance
0.5
0.4
0.3
0.2
0.1
0.0
250
300
350
400
450
500
Wavelength (nm)

(c)

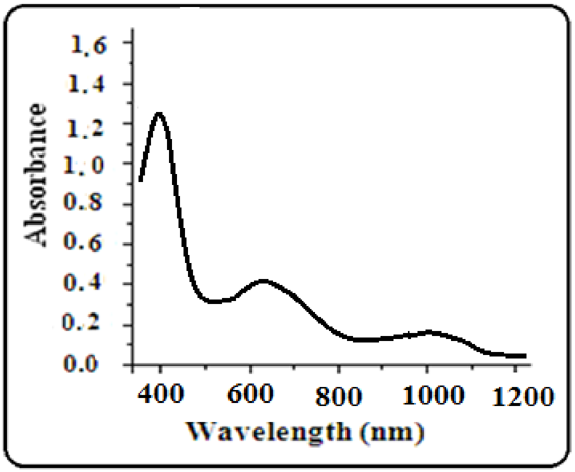
Absorbance
1.6
1.4
1.2
1.0
0.8
0.6
0.4
0.2
0.0
400
600
800
1000
1200
Wavelength (nm)

(d)

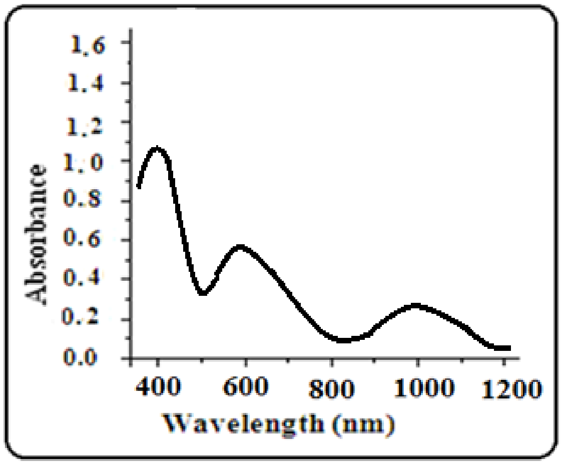
Absorbance
1.6
1.4
1.2
1.0
0.8
0.6
0.4
0.2
0.0
400
600
800
1000
1200
Wavelength (nm)

(e)

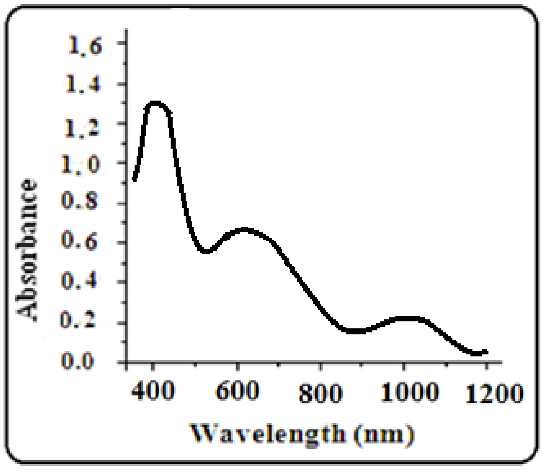
Absorbance
1.6
1.4
1.2
1.0
0.8
0.6
0.4
0.2
0.0
400
600
800
1000
1200
Wavelength (nm)

(f)

Figure S5: (a) Electronic spectrum of HL_1; (b) Electronic spectrum of HL_2; (c) Electronic spectrum of HL_3; (d) Electronic spectrum of C_1; (e) Electronic spectrum of C_2 and (f) Electronic spectrum of C_3.

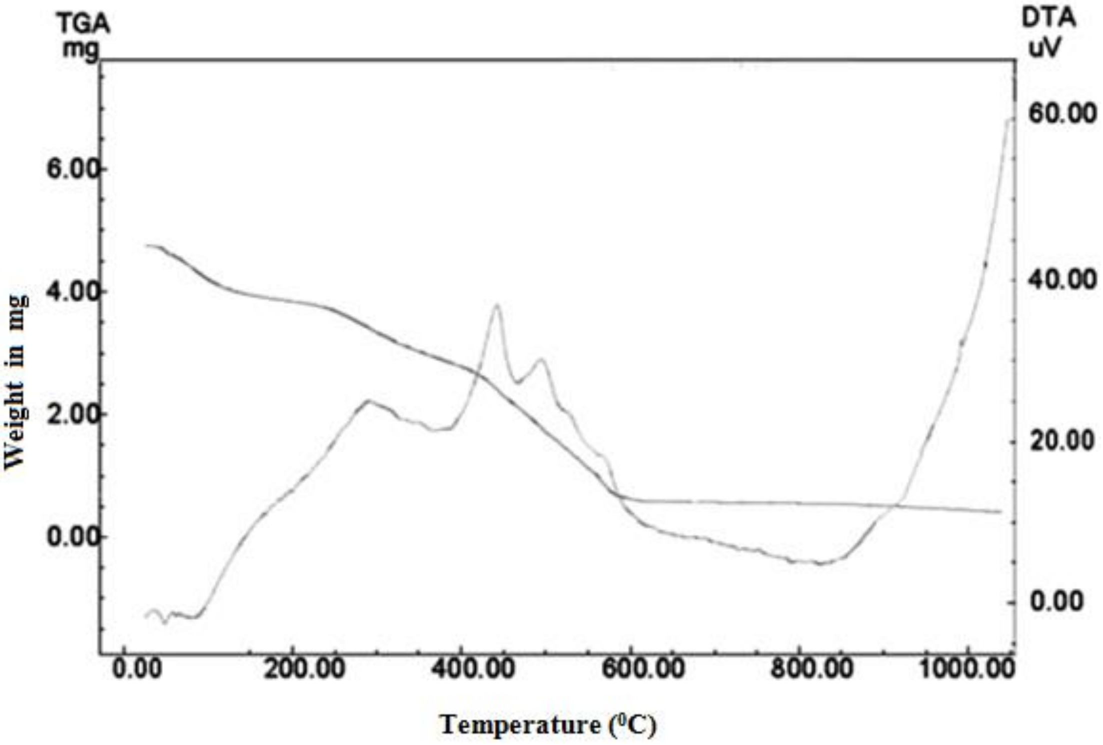

Figure S6: TGA/DTA thermogram of complex C_3.

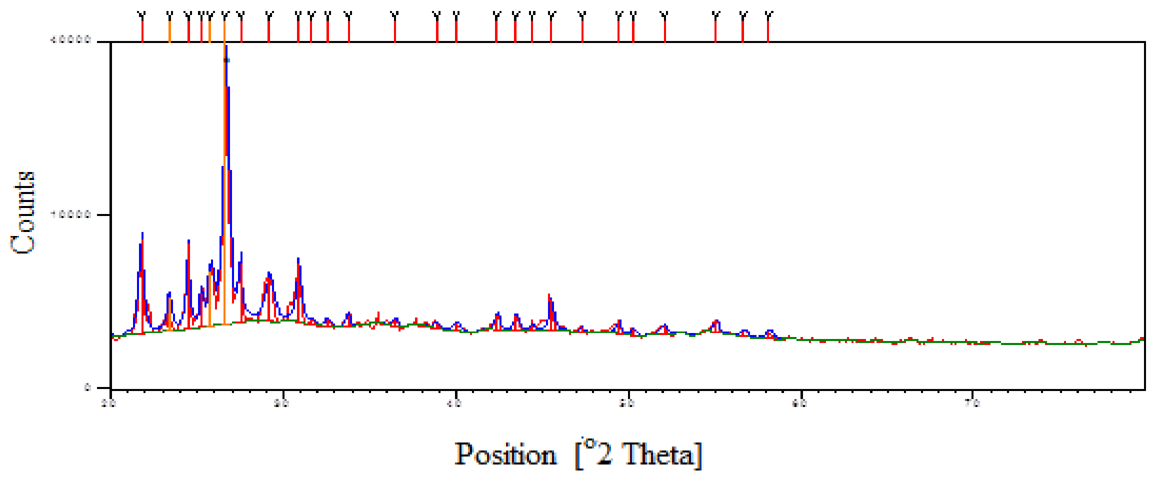
Counts
Position [°2 Theta]

(a)

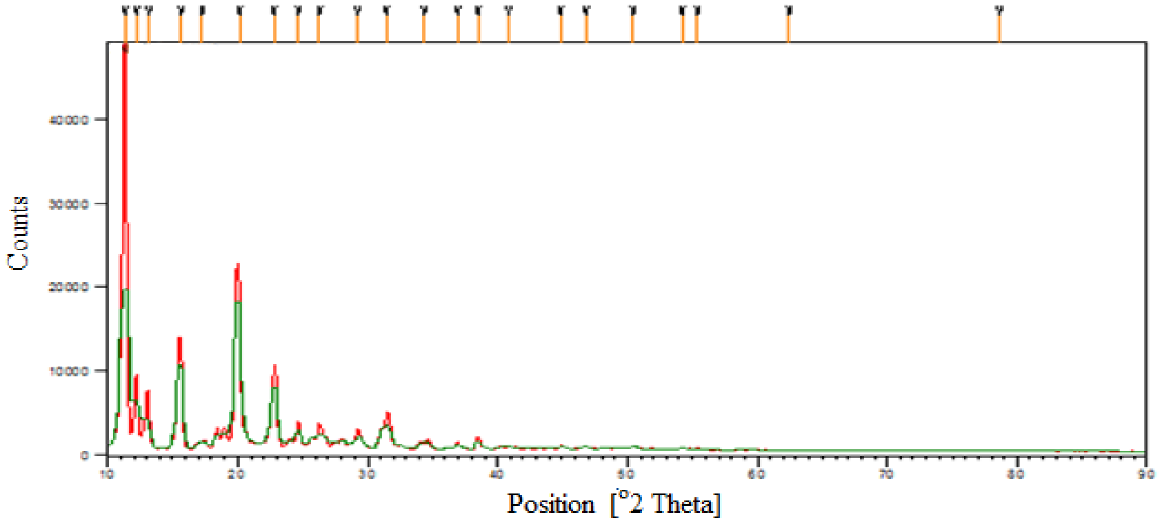
Counts
Position [°2 Theta]

(b)

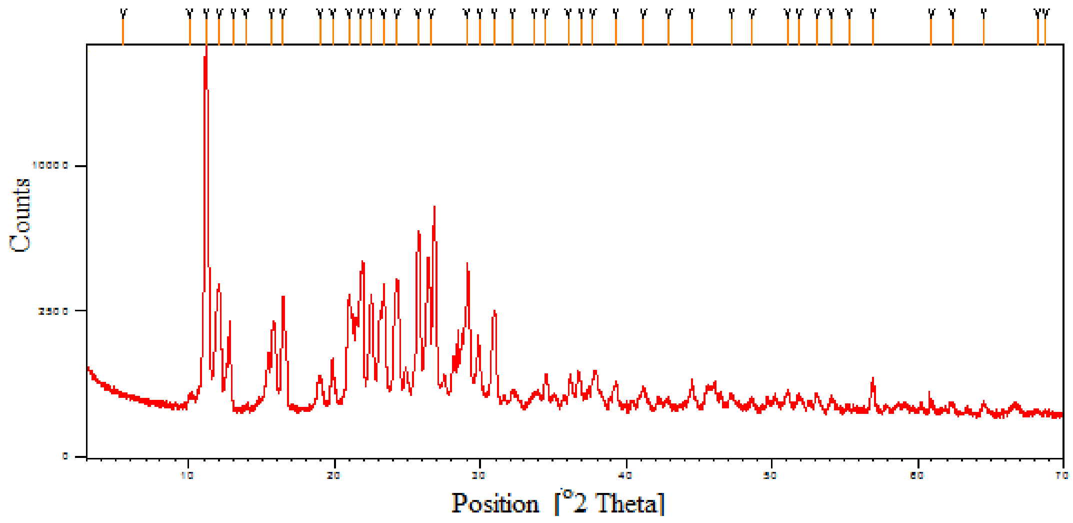
Counts
Position [°2 Theta]

(c)

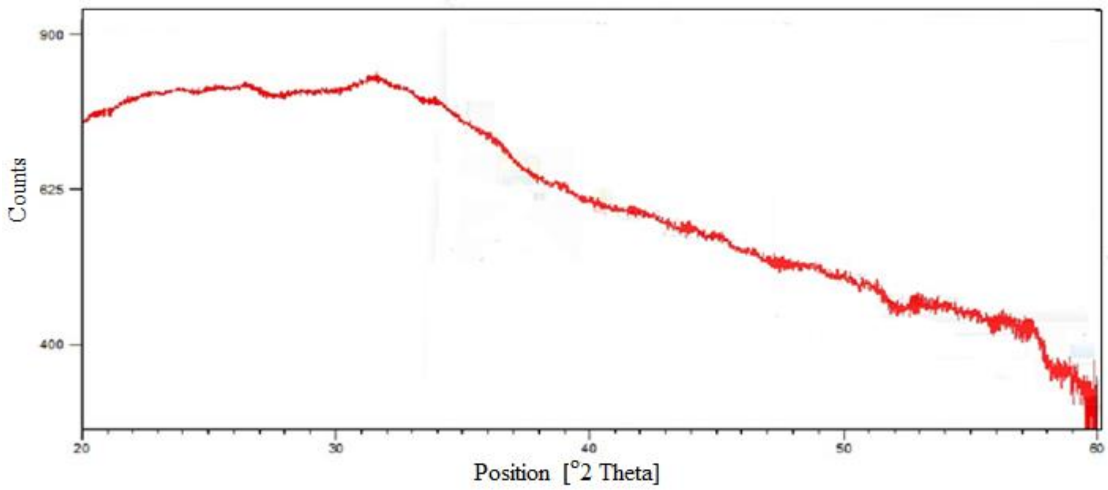

(d)

Figure S7. X-ray diffractogram of (a) HL_1 (b) HL_2, (c) HL_3 and (d) C_3.

Table S1: XRD data of HL_1.

Pos. [°2Th.]	Height [cts]	FWHM [°2Th.]	d-spacing [Å]	Rel. Int. [%]	hkl
21.7677	6477.85	0.2922	4.08292	16.87	020
24.4578	5927.24	0.1948	3.63961	15.44	220
25.2393	1805.91	0.1948	3.52865	4.70	011
26.6576	38391.45	0.2922	3.34404	100	210
27.5059	3839.91	0.1948	3.24280	10.00	211
29.1995	2666.54	0.4871	3.05847	6.95	510
30.8622	3803.80	0.2922	2.89737	9.91	420
45.5015	1526.86	0.2922	1.99350	3.98	240
47.2986	207.64	0.2922	1.92186	0.54	202

Table S2: XRD data of HL_2.

Pos. [°2Th.]	Height [cts]	FWHM [°2Th.]	d-spacing [Å]	Rel. Int. [%]	hkl
11.1580	15275.28	0.3149	7.92995	100.00	001
12.0700	1314.23	0.0010	7.33276	8.60	011
16.4200	1937.85	0.0943	5.39865	12.69	030
20.9880	2481.63	0.3149	4.23283	16.25	211
21.7356	2860.19	0.3149	4.08890	18.72	131
22.4800	1962.72	0.0010	3.95516	12.85	002
23.2793	2452.45	0.3149	3.82114	16.06	012
24.2507	2841.35	0.3149	3.67024	18.60	102
25.6726	4018.52	0.3149	3.47009	26.31	310
29.0414	2689.69	0.3149	3.07477	17.61	150
30.9177	2071.70	0.3149	2.89232	13.56	151

Table S3: XRD data of HL_3.

Po Pos. [°2Th.]	Height [cts]	FWHM [°2Th.]	d-spacing [Å]	Rel. Int. [%]	hkl
11.3588	17760.32	0.6298	7.79024	100	120
12.2408	6121.45	0.0253	7.23094	34.47	001
13.18	6790.74	0.001	6.7176	38.24	210
15.66	28.82	0.001	5.65891	40.21	130
20.18	1273.39	0.001	4.40045	56.05	140
22.773	482.7	0.6298	3.90493	7.17	041
24.72	603.55	0.6298	3.60161	2.72	400
26.2905	424.25	0.6298	3.38992	3.4	022
29.2493	1180.99	0.6298	3.05338	2.39	022
31.4763	1	0.7872	2.84225	6.65	061
38.48	95.94	0.0575	2.33953	7.62	460

Table S4: XRD data of C_3.

Pos. [°2Th.]	Height [cts]	FWHM [°2Th.]	d-spacing [Å]	Rel. Int. [%]	hkl
6.6792	36.38	0.3149	13.23405	7.91	010
7.4380	460.10	0.9210	11.88565	100.00	100
9.6796	14.40	0.3149	9.13756	3.13	011
12.7654	24.48	0.3149	6.93482	5.32	111
18.0112	19.31	0.3149	4.92515	4.20	211
23.7668	222.09	0.1155	3.74386	48.27	301
25.1495	12.52	0.3149	3.54107	2.72	230
28.3192	20.14	0.3149	3.15152	4.38	004
32.0300	261.97	0.0010	2.79437	56.94	204
42.0867	22.58	0.3149	2.14701	4.91	161
53.2203	14.12	0.3149	1.72116	3.07	406
